# In time: celiac disease - some current aspects of epidemiology and research

**DOI:** 10.1016/j.rppede.2016.03.012

**Published:** 2016

**Authors:** Jorge Amil Dias

**Affiliations:** Centro Hospitalar São João, Porto, Portugal

Since the association between gluten intake and celiac disease (CD) was established by Dicke^1^ during World War II, our understanding of the pathophysiology of gluten-sensitive enteropathy has markedly increased, especially with the resources of molecular research. However, although it is clear that gluten intake causes enteropathy and extraintestinal disease in genetically-susceptible individuals, we still lack information on additional factors to triggering mechanisms and disease prevention.

In the 60s, there were a huge tendency to introduce cereals early in infant feeding to prevent iron deficiency and anemia. As a consequence, a marked increase in new cases of celiac disease was observed and the disease seemed to also be related to the type of feeding, which would influence the age of onset of this disease.^2^

In the 80s and 90s, there was an important increase in the incidence of CD in Sweden, causing the famous “Swedish epidemic of Celiac Disease”, which led to several publications and analyses. The most immediate explanation seemed to be related to the age of gluten introduction in the diet and breastfeeding pattern. After implementation of measures to delay the introduction of gluten in infant feeding, there was a marked reduction in the number of new cases.^3^^-^ 5

The analysis of this evolution suggested that the introduction of gluten during breastfeeding could provide protection against the occurrence of the disease. An important multicenter study (PreventCD) compared the introduction of gluten or placebo at 4 months of age during breastfeeding in a group of 900 infants with genetic risk for CD. The study outcome after a five-year follow-up showed that there was no protective effect of breastfeeding during gluten introduction.^6^ Another study involving 553 children evaluated up to two years of age showed that early or late introduction of gluten did not influence the risk of CD, although it influenced the age at its occurrence.^7^ A systematic review achieved the same conclusion, suggesting that the classical recommendation of delaying the introduction of gluten until six months of age currently lacks a scientific basis.^8^

One must conclude that we still do not know what factors can safely reduce the risk of DC in genetically predisposed individuals. Other study hypotheses, such as intrauterine exposure, infections or other environmental factors need to be evaluated in search for the correct answer.^9^

Another very important aspect in the current concept of CD is autoimmunity. It is now well recognized that CD is associated with other autoimmune diseases and the prevalence of CD in patients with type 1 diabetes mellitus (T1DM) is higher than in the general population. The search for serological markers of CD should be carried out in all patients with T1DM. The question becomes even more interesting regarding the analysis of which disease precedes the other and the possibility of influencing the autoimmune progression.^10^ An interesting study was reported in 2015 by Korponay-Szabo: a group of 2,690 schoolchildren underwent screening for CD in 2005. In 2014, a screening for T1DM in the same region was performed, involving 21,724 children. It was then verified that none of the 45 children that had been previously diagnosed with CD and treated had developed T1DM, while the prevalence was 0.93/1,000 among children whose parents had declined the CD screening in 2005. This study, which requires confirmation, suggests that one can modify the development of autoimmunity by screening and early identification of CD. According to the authors, the age of six years seems to be effective for this process (Abstract PA-0054, available at http://journals.lww.com/jpgn/Documents/ESPGHAN%202015%20-%20Abstracts%20JPGN%20FINAL.pdf).

The disclosure of the potential association between gluten intake and diseases has been broadly disseminated in the media. In addition to the “non-celiac sensitivity to gluten”, a good number of famous personalities have announced their decision to adopt a gluten-free diet to lose weight or to feel good. Social networks quickly amplified this trend as a way to be fashionable or healthy (http://glutenull.com/gluten-free-celebrities/). Given this important influence of opinions, many people have currently decided to start a gluten-free diet, even in the absence of a definite CD diagnosis. There is nothing to be disputed regarding the adoption of a gluten-free diet because it is “in fashion”, but there is a real risk in treating CD only temporarily (until it is out of fashion), if the disease is present, and return to the increased risk of complications when a diet without restrictions is resumed. For this reason and because of the inherent risk, it is strongly recommended that health professionals advise their patients to start a gluten-free diet only after being tested with reasonable safety (using, for instance, anti-transglutaminase or anti-endomysial antibodies) and the diagnosis is ruled out or confirmed.

In spite of the great progress in research and knowledge, CD remains a fascinating disease, with an undeniable genetic component, but also environmental factors that are not completely known. In the near future, new developments are expected in the diagnosis and treatment of the disease, which will teach us how to better help our patients.
